# Clinical and biological characteristics associated with bronchial or pulmonary abnormalities on chest CT imaging in patients with systemic mastocytosis

**DOI:** 10.1002/clt2.12387

**Published:** 2024-08-06

**Authors:** Raphael Vallière, Cristina Bulai Livideanu, Thomas Villeneuve, Grégoire Prévot, Laurent L. Reber, Laurent Guilleminault

**Affiliations:** ^1^ Department of Respiratory and Allergic Diseases Toulouse University Hospital Center Toulouse France; ^2^ Department of Dermatology CEREMAST Toulouse Toulouse University Hospital Center Toulouse France; ^3^ Toulouse Institute for Infectious and Inflammatory Diseases (Infinity) Inserm U1291 University of Toulouse CNRS U5051 Toulouse France; ^4^ CRISALIS F‐CRIN/INSERM Toulouse France

To the Editor,

Mastocytosis is a heterogeneous group of diseases characterized by a numerical increase and accumulation of clonal mast cells in various organ systems. In systemic mastocytosis, the severity varies from indolent to aggressive mastocytosis, the latter being associated with a worse prognosis.^1^ Although the lungs are known to be rich in mast cells, comorbid respiratory diseases have only been sporadically suggested as associated with SM in case reports.^2^, ^3^, ^4^ To our knowledge, no data are available on chest CT scans in patients with SM.

The aim of our study is to determine clinical and biological characteristics associated with bronchial or pulmonary abnormalities on chest CT scans in patients with SM.

A retrospective observational study was carried out at Toulouse University Hospital Center from 2003 to 2022 using our mastocytosis registry. All patients with (1) a diagnosis of SM based on bone marrow biopsy according to criteria published elsewhere^1^ and (2) a CT scan image available in their medical record were included. This study was conducted in accordance with French ethics requirements (RC31/17/0095) and the guidelines of the National Commission for Data Protection and Liberties (CNIL number: 2206723 v 0).

Chest CT scans were evaluated by two physicians blinded to the patients' clinical and functional details. The two observers easily established a consensus about the predominant chest CT scan pattern according to the definitions of the Fleischner Society.^5^

Continuous data were expressed as median and interquartile range and categorical data as number of patients and percentages. An increased risk of a predominant pattern on chest CT scan was assessed for aggressive versus indolent SM and serum tryptase ≥20 μg/L versus <20 μg/L. For this analysis, we used a multivariable logistic regression with a calculation of adjusted odds ratios (aORs) with a 95% confidence interval. aORs were adjusted on the following covariates: age, sex and smoking status. We were not able to determine aOR if the CT scan abnormality was absent in the control group.

A total of 103 patients with SM were included in the study (Table 1). Of them, 60.2% were females and the median age was 54 [40–60]. Mastocytosis was indolent in 70 (73%) patients, cKit mutation was found in 91 (85%) patients and median serum tryptase was 15 [30–51] µg/l. A predominant chest CT scan pattern was observed in 36 (35.0%) patients. The lung lesions were as follows: nodules (*n* = 11), emphysema (*n* = 9), bronchiectasis (*n* = 6), bronchial wall thickening (*n* = 6) and interstitial lung diseases (*n* = 4).

**TABLE 1:** Characteristics of the cohort.

	*N* = 103
Age	54 [40–60]
Females	62 (60.2%)
Former‐ or active smokers	48 (46.6%)
Type of mastocytosis	
Indolent	70 (68.0%)
Aggressive	33 (32.0%)
Number of patients with cKit mutation	91 (88.4%)
Serum tryptase (µg/L)	15 [30–51]
Bone marrow tryptase (µg/L) *n* = 89	668 [153–41085]
Number of patients with abnormal CT scan including	36 (35.0%)
Nodules	11 (10.7%)
ILD	4 (3.9%)
Emphysema	9 (8.7%)
Bronchiectasis	6 (5.8%)
Bronchial wall thickening	6 (5.8%)

Abbreviation: ILD, interstitial lung disease.

According to the multivariable analysis, an increased risk of emphysema was observed for aggressive mastocytosis compared to indolent mastocytosis (aOR 5.0 [95% CI: 1.1–27.6]) (Figure 1). An increased risk of bronchial wall thickening was found for serum tryptase ≥20 μg/L (aOR 22.6 [95% CI: 2.4–555.0]). Regarding all other lesions, no increased risk was identified for all groups. We also found no increased risk for a positive methacholine test (data not shown).

**FIGURE 1:**
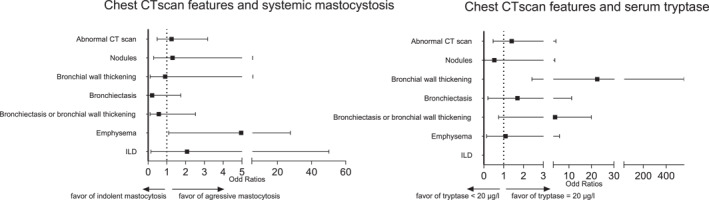
Multivariable logistic regression of chest CT scan patterns for aggressive SM versus indolent SMA, serum tryptase ≥20 μg/L versus < 20 μg/L, bone marrow tryptase ≥50 μg/L versus < 50 μg/L or cKit mutation versus no cKit mutation. Odd ratios (ORs) were adjusted on the following covariates: age, sex and smoking status. If a CT scan pattern was not present in the control group, no aOR was determined. ILD, interstitial lung disease. ILD was defined as ILD with honeycombing and/or bronchiectasis traction.

Respiratory involvement in SM has been poorly described in the literature. In our study, we found that 35% of patients with SM have bronchial or lung lesions on chest CT scan which is systematically performed in our center. In a study conducted in 58 patients with mastocytosis in 1988, Travis et al. found chest radiographic abnormalities in 9 (16%) patients.^6^ Among them, four had focal areas of fibrosis, two had coin lesions, two had scattered areas of fibrosis, one had severe bilateral interstitial fibrosis and one had multiple pulmonary nodules. A lung biopsy was not performed. In the literature, our study is the only one to describe chest CT scan abnormalities in a cohort of patients with SM. Moreover, for the first time, we identified characteristics associated with patterns on chest CT scan in SM.

Our study found that aggressive mastocytosis could be associated with emphysema. In previous case reports, a histological confirmation of mast cell pulmonary infiltration has been observed in patients with SM.^3^, ^4^ It has also been shown that resting mast cells were significantly associated with the clinical features of emphysema.^7^ Aggressive SM may induce a more invasive phenotype of mast cells in lungs leading to alveolar destruction accompanied by the development of emphysema. Although our results seem independent of smoking status, it is likely that smoking habits are an additional risk factor, as it has been observed in a mouse model.

We found that tryptase ≥20 μg/L was associated with an increased risk of bronchial wall thickening. The infiltration of mast cells in the airways could explain our result. Indeed, tryptase levels correlate with mast cell activation. We, therefore, hypothesize that an increased serum tryptase level could correlate with mast cell accumulation and activation in the airways. The mechanism by which this correlation holds still needs to be elucidated. We found no increased risk of bronchial wall thickening with a positive methacholine test in our patients.

Our study has limitations. First, no patients underwent lung biopsies. However, a good correlation between chest CT scans and histological patterns has been widely observed. Secondly, a control group with patients with no mastocytosis were not included. Indeed, our study focused on the chest CT scan characteristics in patients with mastocytosis. For this reason, our results are exclusively obtained from patients with mastocytosis. Finally, due to its retrospective design, we were not able to collect data on respiratory symptoms.

In conclusion, we found that chest CT‐scans identified bronchial or lung lesions in 35% of patients with SM. An increased risk of emphysema was observed for aggressive mastocytosis and bronchial wall thickening for serum tryptase ≥20 μg/l. A prospective study is required to assess the usefulness of chest CT scans in SM.

## AUTHOR CONTRIBUTIONS

**Raphael Vallière**: Conceptualization; investigation; methodology; project administration; validation; visualization; writing – original draft; writing – review & editing; data curation; resources. **Cristina Bulai Livideanu**: Investigation; methodology; validation; visualization; writing – review & editing; writing – original draft; project administration. **Thomas Villeneuve**: Investigation; writing – original draft; methodology; validation; visualization; writing – review & editing; project administration. **Grégoire Prévot**: Investigation; writing – original draft; methodology; validation; visualization; writing – review & editing; project administration. **Laurent L. Reber**: Investigation; writing – original draft; methodology; validation; visualization; writing – review & editing; project administration. **Laurent Guilleminault**: Conceptualization; investigation; writing – original draft; methodology; validation; visualization; writing – review & editing; formal analysis; project administration; supervision; data curation; resources.

## CONFLICT OF INTEREST STATEMENT

Laurent Guilleminault has been an investigator in clinical trials for AstraZeneca, MSD and Novartis, reports grants or consultation fees from AstraZeneca, GlaxoSmithKline, Novartis and Sanofi‐Regeneron, and consultation fees from Bayer, Chiesi and MSD, none of which is related to the work submitted. Thomas Villeneuve declares consultation fees from Boehringer Ingelheim and Mauna Kea Technologies that are not related to the work submitted. Cristina Bulai Livideanu has been an investigator in clinical trials for Abbvie, ABScience, BluePrint Medicine, Cogent, Janssen, Lilly, Novartis and Pfizer and reports consultation fees from Abbvie, Blueprint Medicine, Janssen, Lilly and Novartis, none of which is related to the work submitted. Laurent L. Reber is or recently was a speaker and/or advisor for and/or has received research funding from Argenx, Novartis, Ceva and Neovacs, not related to the work submitted.

## CLINICAL IMPLICATIONS BOX

We found that 35% of patients with SM have bronchial or pulmonary abnormalities on chest CT scans. An increased risk of emphysema was observed for aggressive mastocytosis and bronchial wall thickening for serum tryptase ≥20 μg/L.

## Data Availability

Data will be available on request.
